# SARS-CoV-2 Nucleocapsid Protein Targets RIG-I-Like Receptor Pathways to Inhibit the Induction of Interferon Response

**DOI:** 10.3390/cells10030530

**Published:** 2021-03-02

**Authors:** Soo Jin Oh, Ok Sarah Shin

**Affiliations:** BK21 Graduate Program, Department of Biomedical Sciences, College of Medicine, Korea University Guro Hospital, Seoul 08308, Korea; sjooooh@gmail.com

**Keywords:** coronavirus disease 2019, SARS-CoV-2 N protein, antiviral immune response, interferon, RIG-I like receptors

## Abstract

Severe acute respiratory syndrome coronavirus 2 (SARS-CoV-2) is the causative agent of the coronavirus disease 2019 (COVID-19) that has resulted in the current pandemic. The lack of highly efficacious antiviral drugs that can manage this ongoing global emergency gives urgency to establishing a comprehensive understanding of the molecular pathogenesis of SARS-CoV-2. We characterized the role of the nucleocapsid protein (N) of SARS-CoV-2 in modulating antiviral immunity. Overexpression of SARS-CoV-2 N resulted in the attenuation of retinoic acid inducible gene-I (RIG-I)-like receptor-mediated interferon (IFN) production and IFN-induced gene expression. Similar to the SARS-CoV-1 N protein, SARS-CoV-2 N suppressed the interaction between tripartate motif protein 25 (TRIM25) and RIG-I. Furthermore, SARS-CoV-2 N inhibited polyinosinic: polycytidylic acid [poly(I:C)]-mediated IFN signaling at the level of Tank-binding kinase 1 (TBK1) and interfered with the association between TBK1 and interferon regulatory factor 3 (IRF3), subsequently preventing the nuclear translocation of IRF3. We further found that both type I and III IFN production induced by either the influenza virus lacking the nonstructural protein 1 or the Zika virus were suppressed by the SARS-CoV-2 N protein. Our findings provide insights into the molecular function of the SARS-CoV-2 N protein with respect to counteracting the host antiviral immune response.

## 1. Introduction

A newly emerging infectious disease COVID-19 is caused by SARS-CoV-2, and was first reported in Wuhan, China [1]. In March 2020, the COVID-19 outbreak was declared as a global public health emergency by the World Health Organization. Clinical manifestations of SARS-CoV-2 infection in humans are characterized by a wide spectrum of symptoms that range in severity from mild fever, cough, and dyspnea to neurological complications, respiratory failure, and death [2]. The absence of highly efficacious antiviral drugs, in addition to the high transmissibility of COVID-19, highlight the urgent need to establish a better understanding of the host–pathogen interaction of SARS-CoV-2 to enable the development of new therapies and vaccines.

The genome of SARS-CoV-2 is approximately 30 kilobases in length and encodes at least six open-reading frames (ORFs). At the 5′ end, two large genes, ORF1a and ORF1b, encode 16 non-structural proteins (NSP1–NSP16), whereas other ORFs located at the 3′ end encode the following four essential structural proteins: spike (S), envelope (E), membrane (M), nucleocapsid (N), and accessory proteins [3,4]. Although the current mortality rate associated with SARS-CoV-2 infection is not as high as that of SARS-CoV-1 or MERS-CoV, the presence of asymptomatic patients and significant viral shedding during the incubation period that account for the high transmission rate of SARS-CoV-2 pose a significant health threat. The ability of SARS-CoV-2 to evade and suppress the early immune response during the early stage of infection may explain how the virus persists to spread worldwide. However, the role of SARS-CoV-2 proteins in suppressing antiviral immunity remains largely unexplored.

Type I and III IFNs are triggered by viral recognition by innate immune sensors and function as the first line of defense against viruses [5]. Despite these host antiviral immune strategies, coronaviruses have evolved unique evasion mechanisms—such as interfering with viral sensing, IFN production and signaling, and interferon-stimulated gene (ISG) expression and functions [6,7]. Recent findings suggest that SARS-CoV-2, when compared to other respiratory viruses such as the influenza virus, is a poor inducer of type I IFN response in vitro and in animal models [8,9,10]. Currently, impaired or delayed IFN response in the early phase of COVID-19 disease is believed to be associated with the disease severity in patients with COVID-19 who display enhanced cytokine production and inflammation [11].

In this study, we report that the nucleocapsid protein (N) of SARS-CoV-2 is a potent IFN antagonist. SARS-CoV-2 N expression resulted in the attenuation of IFNs and downstream ISG activation via interfering with RIG-I and TRIM25 interaction. In addition, the production of both type I and III IFNs induced by the influenza virus lacking nonstructural protein 1 (IFV-delNS1) or Zika virus (ZIKV) was diminished by the SARS-CoV-2 N protein. Our findings contribute new insights into the mechanism underlying N protein-mediated suppression of IFN signaling associated with the innate immune evasion of SARS-CoV-2.

## 2. Materials and Methods

### 2.1. Cells, Viruses and Reagents

HEK293T, HeLa, and A549 cells were obtained from American Type Culture Collection (ATCC; Manassas, VA, USA). A549 cells were cultured in RPMI 1640 medium (Corning Mediatech, Corning, NY, USA) with 10% fetal bovine serum (FBS; Corning Mediatech) and 1% penicillin-streptomycin. HEK293T and Hela cells were cultured in DMEM medium (Corning Mediatech) with 10% FBS and 1% penicillin-streptomycin.

Human influenza virus A/Puerto-Rico/8/34 (H1N1) PR8 lacking non-structural protein (NS1) open reading frame (IFV-delNS1) was previously reported [12]. A standard plaque assay was performed to determine viral titers in MDCK cells [13,14]. ZIKV MR766 (African lineage) was purchased from ATCC and propagated in Vero cells, as previously described [15,16,17].

Poly I:C (Sigma-Aldrich, St. Louis, MO, USA) was transfected at 10 μg/mL with Lipofectamine 2000 transfection reagent (Invitrogen, Carlsbad, CA, USA). Recombinant human IFN-α and IFN-β proteins were purchased from PeproTech (Rocky Hill, NJ, USA).

### 2.2. Plasmid Transfection 

Full length RIG-I-FLAG and RIG-IC-FLAG (CARD domain of RIG-I)-encoding plasmids were gifted by Dr. Takashi Fujita (Kyoto University, Japan). Empty vector-green fluorescent protein (EV-GFP), SARS-CoV-2 N-GFP, melanoma differentiation-associated protein 5 (MDA5)-HIS, tripartite motif containing 25 (TRIM25)-HA, and TBK1-MYC were obtained from Sino Biological (Beijing, China). ZIKV NS5 plasmid was described previously [15]. Full length mitochondrial antiviral signaling protein (MAVS)-MYC was provided by Dr. Jewook Yu (Yonsei University School of Medicine, Seoul, Korea). Full-length plasmids encoding IRF3 and IRF3 (5D), a constitutively active mutant form of IRF3, were obtained from Dr. Moon Jung Song at Korea University College of Life Sciences and Biotechnology. For transient transfection, plasmids were mixed with Lipofectamine 2000 transfection reagent (Invitrogen) in Opti-MEM, and the mixture was incubated for 20 min at room temperature. After 24 h, protein was extracted to determine transfection efficiency by Immunoblot analysis.

### 2.3. Luciferase Reporter Assay 

HEK293T or A549 cells were plated in 96-well plates and transiently transfected with mixture of plasmids using Lipofectamine 2000 transfection reagent next day. Empty control plasmid was used to ensure that each transfection well contains the same amount of total DNA. The following luciferase reporter plasmids were used: IFN-β, IFN-λ1, IFN-λ2 (kindly gifted by Dr. Tomozumi Imamichi, Frederick National Laboratory for Cancer Research, Frederick, MD, USA), 5X IFN-stimulatory regulatory element (ISRE), IP-10, and NF-κB promoters in combination with EV-GFP or SARS-CoV-2 N-GFP. As an internal control, 10 ng of Renilla luciferase reporter plasmid was transfected simultaneously. At 24 h post-transfection, the luciferase activity was detected with Dual-glo luciferase reporter assay system (Promega, Madison, WI, USA) using Varioskan™ LUX multimode microplate reader (Thermo Scientific, Waltham, MA, USA). 

### 2.4. Confocal Microscopy

Cells growing on coverslips were transfected with various plasmids. Mito-dsRED plasmid (a kind gift of Dr. Woong Sun, Korea University School of Medicine) was used to indicate mitochondria. Cells were washed with phosphate-buffered saline (PBS), fixed in 4% paraformaldehyde, and permeabilized with 0.1% Triton X-100 as described previously [17]. Cells were then blocked and incubated with the indicated primary antibodies followed by incubation with a fluorescent secondary antibody. The cells were stained with DAPI and the images were examined using a confocal microscope (LSM900; Carl Zeiss, Oberkochen, Germany).

### 2.5. Real-Time Reverse Transcriptase-Quantitative Polymerase Chain Reaction (RT-qPCR)

Total RNA from harvested cells were extracted with TRIzol reagent (Invitrogen). cDNA synthesis was performed using ImProm-II Reverse Transcription System (Promega). Quantitative real-time PCR was performed with Power SYBR Green Master Mix (Invitrogen) using QuantStudio 6 Flex Real-time PCR system (Thermo Fisher Scientific, Waltham, MA, USA). 

### 2.6. Immunoblot Analysis

RIPA buffer (Sigma-Aldrich) plus a protease and phosphatase inhibitor cocktail (Roche, Basel, Switzerland) was used to lyse cells as previously described [18]. Proteins were loaded onto SDS-PAGE gels, transferred onto polyvinylidene difluoride membranes, and blocked with 5% skim milk in Tris-buffered saline supplemented with 0.1% Tween-20 (TBS-T) for 1 h at room temperature. The membranes were incubated overnight with primary antibodies at 4 °C. The following antibodies were used; Anti-FLAG tag (Wako), Anti-MYC tag (Abcam), Anti-GFP (Santa Cruz), Anti-Tubulin or β-actin antibodies (Abgent). Anti-pSTAT1/STAT1, Anti-HA tag, Anti-HIS tag, Anti-MYC tag, Anti-MDA5, Anti-RIG-I, Anti-pTBK1/TBK1, Anti-pIRF3/IRF3, Anti-MxA, Anti-OAS1, and Anti-RNaseL antibodies were from Cell Signaling Technology. Anti-SARS-CoV/SARS-CoV-2 Nucleocapsid Antibody (Sino Biological), while Anti-IFV NP antibody and Anti-ZIKV E antibody were purchased from GeneTex. The blots were washed with TBST for three times and incubated with HRP-conjugated secondary antibodies (Cell Signaling Technology, Danvers, MA, USA). Proteins were visualized with ECL Western Blotting Substrate (Thermo Fisher Scientific, Waltham, MA, USA). Densitometry to quantify immunoblot bands was performed using ImageJ software.

### 2.7. Co-Immunoprecipitation

Cells were harvested and lysed with Pierce IP Lysis Buffer (Thermo Fisher Scientific, Waltham, MA, USA) plus s complete protease inhibitor cocktail (Roche, Basel, Switzerland). Surebeads Protein A Magnetic Beads (Bio-Rad Laboratories, Hercules, CA, USA) were incubated with indicated antibodies for 10 min at room temperature. The beads were added to samples and incubated overnight at 4 °C. Immunoprecipitates were washed and eluted in SDS sample buffer for subsequent immunoblot analysis.

### 2.8. Cytokine Secretion Measurements by Enzyme-Linked Immunosorbent Assay (ELISA)

The level of IFN-λ1/3 secretion in culture supernatants were measured using ELISA kits (R&D Systems, Minneapolis, MN, USA) according to the manufacturer’s recommendations.

### 2.9. Statistical Analysis 

All experiments were performed at least three times and the data are shown as the mean + standard deviation. Statistical analyses were performed using Prism 7.0 software (Graphpad Inc., San Diego, CA, USA) and *p* values were calculated using Mann–Whitney test or Student’s *t* test. 

## 3. Results

### 3.1. SARS-CoV-2 N Attenuates IFN Promoter Activities and Signaling

**Figure 1:**
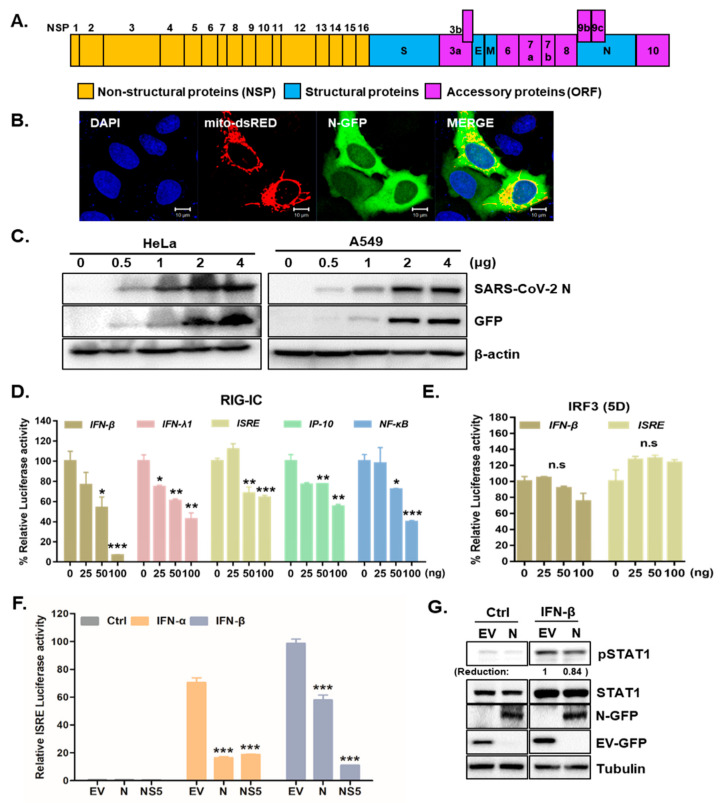
SARS-CoV-2 N is a potent interferon antagonist. (**A**) Schematic view of SARS-CoV-2 genome is shown. (**B**) HeLa cells were transfected with SARS-CoV-2 N-GFP-encoding and mito-dsRED plasmids. Scale bar = 10 μm. (**C**) HeLa and A549 cells transfected with increasing amounts of SARS-CoV-2 N-GFP-encoding plasmids were immunoblotted with with the indicated antibodies to determine transfection efficiency. (**D**,**E**) HEK293T cells expressing RIG-IC, or IRF3 (5D) were co-transfected with firefly luciferase reporter plasmids encoding IFN-β, IFN-λ1, ISRE, IP-10, and NF-κB promoter together with increasing amount of SARS-CoV-2 N. The EV-GFP plasmid was used to ensure that each sample contains the same amount of total DNA plasmids. * *p* < 0.05; ** *p* < 0.01; *** *p* < 0.001, non-significant (n.s) versus EV-GFP-transfected cells (**F**) Recombinant IFN-α (50 ng/mL) or IFN-β (50 ng/mL) protein was added to the cells following the transfection with 100 ng of EV-GFP (EV), SARS-CoV-2 N (N) or ZIKV NS5 (NS5) encoding plasmids. Mean ± SD of three technical replicates are shown. *** *p* < 0.001 versus EV-GFP-transfected cells. (**G**) Immunoblot results of IFN-β-induced phosphorylation of STAT1 are shown. The images are representative of three independent experiments. Numbers indicate quantitative densitometric analyses of blot using ImageJ software.

The genome of SARS-CoV-2 contains non-structural and structural proteins, such as the spike (S), envelope (E), membrane (M), nucleocapsid (N), and accessory proteins [19], as described in Figure 1A. The SARS-CoV N protein is essential for encapsulating viral genomic RNA and suppressing antiviral IFN pathways [20,21]. To investigate the role of SARS-CoV-2 N in modulating the host innate immune response, SARS-CoV-2 N-GFP plasmids were transiently transfected into HeLa or A549 cells. The transfection efficiency of SARS-CoV-2 N-GFP plasmids was detected by both confocal microscopy and immunoblot analysis (Figure 1B,C). To determine whether SARS-CoV-2 N suppressed IFNs, we conducted luciferase assays to measure the effect of SARS-CoV-2 N on IFN signaling activities via IFN-β and ISRE promoter activation. The luciferase reporter assays revealed that overexpression of SARS-CoV-2 N in HEK293T cells reduced both IFN-β and ISRE-dependent IFN signaling mediated by RIG-I activation in a dose-dependent manner (Figure 1D). Interestingly, both IFN-β and ISRE-dependent IFN signaling was not affected by IRF3(5D) transfection (Figure 1E), suggesting that SARS-CoV-2 N may play a role in downstream of TBK1. We also measured the effect of SARS-CoV-2 N overexpression on IFN-α induced signaling through ISRE promoter activation via luciferase reporter assay. As a positive control, ZIKV nonstructural protein (NS5) was used as an IFN signaling antagonist. Figure 1F shows that IFN-α or IFN-β dependent ISRE and activation was significantly reduced by SARS-CoV-2 N, similar to that by ZIKV NS5 protein. These results suggest that SARS-CoV-2 N blocks ISRE promoter activation induced by either IFN-α or IFN-β, emphasizing the role of SARS-CoV-2 N in counteracting multiple steps of IFN-mediated antiviral pathway. Moreover, similar to previous finding by Mu et al. [22], our result also show that IFN-induced phosphorylation of STAT1 can be inhibited by SARS-CoV-2 N protein (Figure 1G).

**Figure 2:**
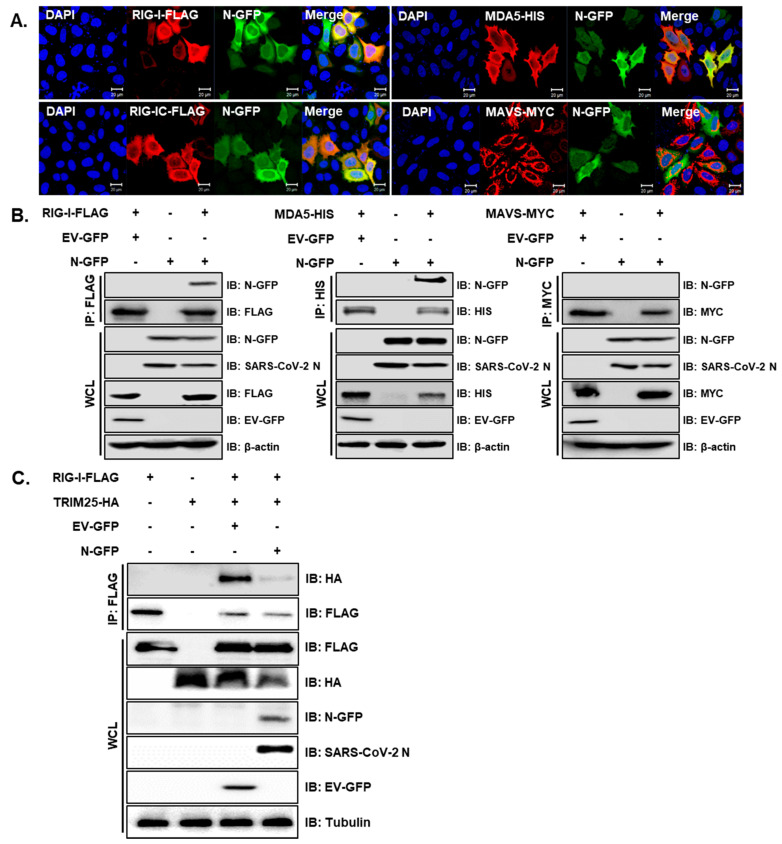
SARS-CoV-2 N interacts with genes involved in the RLR signaling pathway and interferes RIG-I interaction with TRIM25. (**A**) HeLa cells were transiently transfected with the following plasmids: RIG-I-FLAG, RIG-IC-FLAG, MDA5-HIS and MAVS-MYC along with SARS-CoV-2 N-GFP-encoding plasmids. Cells were fixed in paraformaldehyde and permeabilized with 0.1% Triton X-100. The images are representative of three independent experiments. Scale bar = 20 μm. (**B**) HEK293T cells in 6-well plates were transfected with RIG-I-FLAG or MAVS-MYC plasmids along with empty vector with GFP tag (EV-GFP) or SARS-CoV-2 N-GFP (N-GFP) plasmids. After 24 h, cells were lysed and immunoprecipitated with indicated antibodies. Whole cell lysates (WCL) and immunoprecipitates were analyzed by immunoblot analysis. (**C**) HEK293T cells transfected with plasmids encoding RIG-I-FLAG or TRIM25-HA along with EV-GFP or N-GFP-encoding plasmids were lysed and subjected to immunoprecipitation with anti-FLAG tag antibody. The cell lysates and immunoprecipitates were analyzed by immunoblot analysis using indicated antibodies.

To evaluate which proteins cellularly co-localize with SARS-CoV-2 N protein in humans, Hela cells were transiently transfected with RIG-I, MDA5 or MAVS, along with the SARS-CoV-2 N-GFP plasmid. The interactions between full-length RIG-I or MDA5 and SARS-CoV-2 N-GFP were detected by confocal microscopy and verified with co-immunoprecipitation assay (Figure 2A,B). Notably, the SARS-CoV-2 N protein did not appear to interact with MAVS. Reciprocal immunoprecipitation experiment further confirmed that RIG-I and MDA5 immunoprecipitate with SARS-CoV-2 N protein, while MAVS does not (Supplementary Figure S1). Hu et al. previously reported that SARS-CoV-1 N protein interfered with the interaction between TRIM25 and RIG-I, thereby inhibiting TRIM25-mediated RIG-I ubiquitination and activation [23]. Therefore, we performed a co-immunoprecipitation assay to determine the role of SARS-CoV-2 N. The interaction between RIG-I and TRIM25 was subsequently attenuated by the co-expression of the SARS-CoV-2 N protein, as shown in Figure 2C. These results collectively indicate that the SARS-CoV-2 N protein can specifically interact with genes involved in RIG-I-like receptor (RLR) signaling pathway and inhibit the direct interaction between TRIM25 and RIG-I.

### 3.2. SARS-CoV-2 N Blocks Poly I:C-Triggered Activation of IFN Pathway via Abrogating the Interaction between TBK1 and IRF3

**Figure 3:**
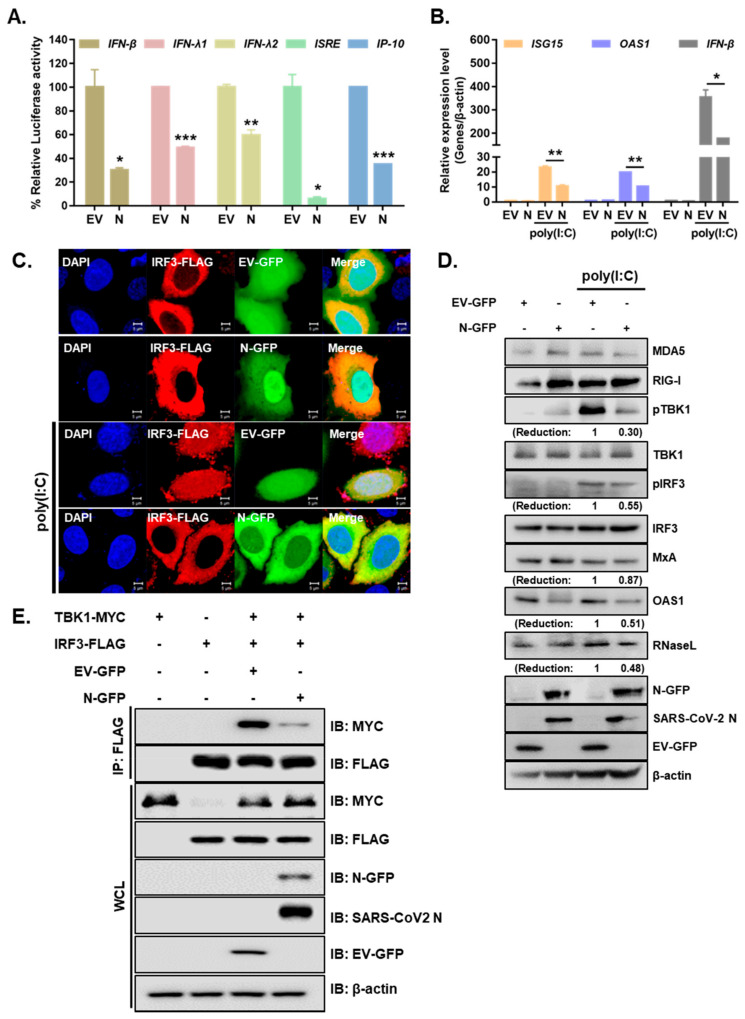
SARS-CoV-2 N inhibits the activation of IFN production and signaling in response to poly I:C treatment. (**A**) Luciferase activities of A549 cells transfected with 100 ng of EV-GFP (EV) or SARS-CoV-2 N-GFP (N) plasmids along with IFN-β, IFN-λ1, IFN-λ2, ISRE, and IP-10 promoter luciferase plasmids for 24 h and treated with poly (I:C) (10 µg/mL) for 6 h (mean ± SD, n = 3). * *p* < 0.05; ** *p* < 0.01; *** *p* < 0.001 versus EV-GFP-transfected cells. (**B**) A549 cells expressing either EV or N were transfected with poly (I:C) (10 µg/mL) for 6 h. RT-qPCR analysis of *ISG15, OAS1*, and *IFN-β* mRNAs is shown (mean ± SD, n = 3). * *p* < 0.05; ** *p* < 0.01 versus EV-GFP-transfected cells.(**C**) A549 cells were co-transfected with IRF3-FLAG and EV-GFP or N-GFP plasmids. After 24 h, cells were treated with poly I:C (10 µg/mL) for 2 h and fixed to observe the subcellular localization of IRF3. Scale bar = 5 μm. (**D**) A549 cells were transiently transfected with EV-GFP or N-GFP plasmids. After 24 h, cells were transfected with poly (I:C) (10 µg/mL) for 2 h. Cell lysates were collected for immunoblotting with the indicated antibodies. Numbers indicate quantitative densitometric analyses of blot using ImageJ software. (**E**) HEK293T cells were transfected with plasmids encoding IRF3-FLAG and TBK1-MYC along with EV-GFP or N-GFP for 24 h. Cell lysates were subjected to immunoprecipitation with an anti-FLAG tag antibody, followed by immunoblot analysis with indicated antibodies.

Next, we examined whether the SARS-CoV-2 N protein is involved in regulating the IFN-β signaling pathway that is mediated by poly (I:C), an analog of double-stranded RNA. A549 cells expressing either EV-GFP or SARS-CoV-2 N-GFP were treated with poly I:C for 6 h. The expression levels of proteins involved in RLR pathways were measured by immunoblot analysis. As shown in Figure 3A, IFN-β, IFN-λ1, IFN-λ2, ISRE and IP-10 promoter activities mediated by poly I:C treatment in A549 cells were significantly reduced by SARS-CoV-2 N overexpression. Further RT-qPCR experiments were performed to determine whether the overexpression of SARS-CoV-2 N inhibited poly (I:C)-triggered activation of ISG transcription in A549 cells. We observed that poly (I:C)-triggered transcription of the *ISG15*, *OAS1*, and *IFN-β* genes was markedly inhibited in SARS-CoV-2 N-overexpressing A549 cells compared with that of EV-transfected cells (Figure 3B). Next, we determined whether SARS-CoV-2 N affects the nuclear translocation of IRF3, which is a key step in IRF3-mediated activation of type I IFN genes. Confocal microscopy analysis revealed that nuclear import of IRF3 after poly (I:C) stimulation was significantly inhibited in SARS-CoV-2 N-overexpressing A549 cells compared with that in EV-transfected cells (Figure 3C). In addition, poly (I:C) treatment of A549 cells led to elevated protein levels of RLRs, such as MDA5and RIG-I, or downstream molecules of RLRs, such as phospho-TBK1 and phospho-IRF3, in EV-transfected cells as compared to those in SARS-CoV-2 N-GFP-transfected cells (Figure 3D). Poly (I:C)-triggered phosphorylation of TBK1 and IRF3, which are hallmarks of virus-triggered activation of IFN pathways, was markedly lower in cells overexpressing SARS-CoV-2 N compared to that in EV-transfected cells. Moreover, the induction of ISGs (MxA, OAS1 RNaseL) was also attenuated upon SARS-CoV-2 N-GFP expression in A549 cells. Additionally, the co-immunoprecipitation assay revealed that SARS-CoV-2 N reduced the interaction between TBK1 and IRF3 (Figure 3E). These results suggest that SARS-CoV-2 N interferes with TBK1-induced IRF3 activation by abolishing the interaction between TBK1 and IRF3.

### 3.3. Overexpression of SARS-CoV-2 N Inhibits Virus-Triggered Activation of Type I and III IFN Production

**Figure 4:**
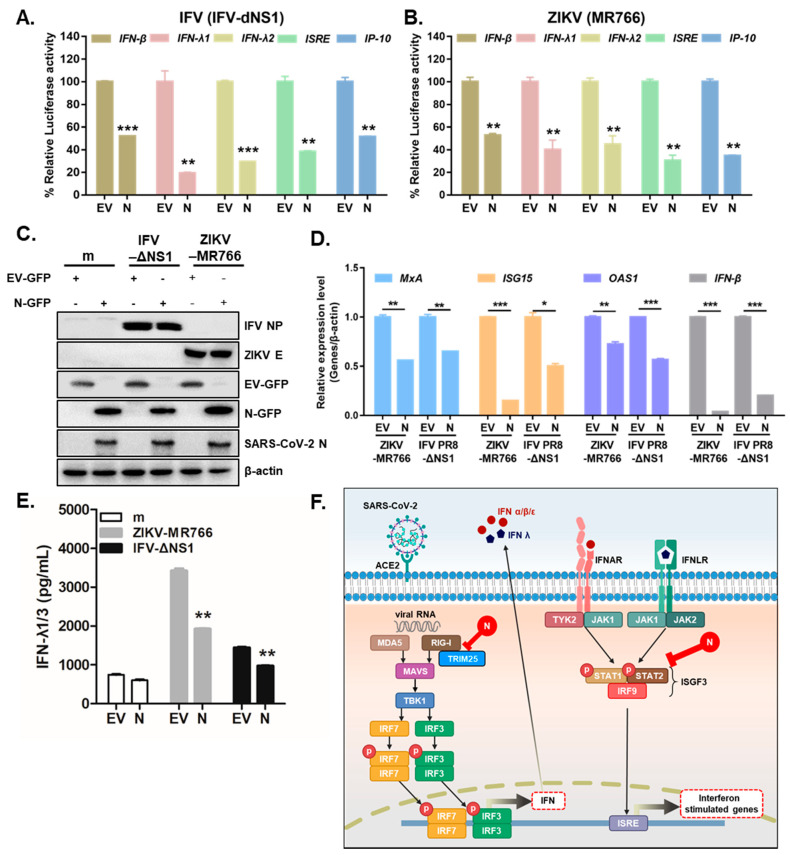
SARS-CoV-2 N markedly attenuates RNA virus-triggered IFN production. (**A**,**B**) Luciferase activities of A549 cells transfected with IFN-β, IFN-λ1, IFN-λ2, ISRE, and IP-10 promoter luciferase plasmids together with expression vector for RIG-I, along with 100 ng of EV-GFP (EV) or SARS-CoV-2 N-GFP (N) expression plasmids. At 24 h after transfection, the cells were infected with IFV-delNS1 (IFV-dNS1) (A) or ZIKV (MR766) (**B)** at multiplicity of infection (MOI) = 1 for 6 h (mean ± SD, n = 3). ** *p* < 0.01; *** *p* < 0.001 versus EV-GFP-transfected cells. (**C**) A549 cells were transiently transfected with EV-GFP or SARS-CoV-2 N-GFP expression plasmids. After 24 h, cells were infected with mock (m), ZIKV (MR766), or IFV-delNS1 (MOI = 1). (**D**) RT-qPCR reveals mRNA levels of *MxA, IFN-β, ISG15*, and *OAS1* following viral infection. * *p* < 0.05; ** *p* < 0.01; *** *p* < 0.001 versus EV-GFP-transfected cells. (**E**) IFN-λ1/3 secretion levels were measured by ELISA assays. ** *p* < 0.01 versus EV-GFP-transfected cells. (**F**) Schematic representation of how SARS-CoV-2 N protein interferes with IFN induction and signaling.

Previous studies have suggested that several RNA viruses, such as ZIKV (MR766) or influenza virus lacking NS1 (IFV-delNS1), trigger antiviral immune responses via the induction of IFNs and downstream IFN-stimulated genes in A549 cells [16,24]. To determine whether SARS-CoV-2 N modulates RNA virus-triggered IFN expression, A549 cells were transiently transfected with the following luciferase plasmids: IFN-β, IFN-λ1, IFN-λ2, ISRE, and IP-10. SARS-CoV-2 N inhibited the IFV-delNS1-induced induction of the IFN-β, IFN-λ1, IFN-λ2, ISRE, and IP-10 promoter activities mediated by RIG-I activation in A549 cells (Figure 4A).Similar results were observed in response to ZIKV (MR766) infection. (Figure 4B). To ascertain whether SARS-CoV-2 N would affect the virus-triggered activation of IFNs and ISGs, A549 cells were transiently transfected with SARS-CoV-2 N and infected with either ZIKV (MR766) or IFV (IFV-delNS1), and ISG mRNA levels were measured by RT-qPCR. Immunoblot analysis confirmed efficient EV-GFP or N-GFP transfection in A549 cells (Figure 4C). Figure 4D reveals that the mRNA levels of *MxA, IFN-β, ISG15,* and *OAS1* were significantly reduced following SARS-CoV-2 N expression in ZIKV or IFV-infected cells. Recently, type III IFNs have been studied extensively in the context of ZIKV infection because they play a key role in mounting antiviral responses in epithelial cells [15]. Thus, we also examined whether SARS-CoV-2 N protein had any effect on type III IFN production. The ELISA assay showed a significant increase in IFN-λ1/3 in response to ZIKV or IFV. However, the induction of type III IFN secretion levels was detected significantly lower following ZIKV (MR766) or IFV (IFV-delNS1) infection in SARS-CoV-2 N-expressing cells compared with those in EV-transfected cells (Figure 4E). These data suggest SARS-CoV-2 N’s ability to suppress both type I and III IFN signaling in response to ZIKV (MR766) or IFV (IFV-delNS1) infection.

## 4. Discussion

After emerging in December 2019, COVID-19 has transformed into a pandemic, likely owing to highly infectious nature and its efficient strategies for evading antiviral immunity. It is recently becoming clear that SARS-CoV-2, similar to SARS-CoV-1, encodes multiple proteins that facilitates the virus to evade and suppress antiviral IFNs. For example, a recent study demonstrated that non-structural protein (nsp) 13, nsp14, nsp15, and orf6 of SARS-CoV-2 act as potent inhibitors of IFN signaling pathways, thereby suppressing IFN gene expression and production [25]. Furthermore, Mu et al. recently reported association of SARS-CoV-2 N protein with STAT1 and STAT2, indicating N protein’s ability to further interfere with phosphorylation and nuclear translocation of STATs [22]. In addition to these studies, here we present possible mechanisms utilized by the SARS-CoV-2 N protein to evade IFN-mediated response.

The nucleocapsid protein (N) of coronaviruses is known to have multifunctional RNA binding properties required for the process of viral replication, transcription and translation [26,27]. In addition to its structural role in viral RNA binding and assembly, N protein has shown to modulate host-pathogen interactions, such as controlling apoptosis, actin reorganization and host cell cycle progression [28,29,30]. Moreover, recent studies highlighted inhibitory roles of SARS-CoV-2 N protein, especially in terms of host immune evasion and IFN antagonist [22,31]. Previous studies have shown that both SARS-CoV-1 and MERS-CoV fail to elicit robust IFN expression. In particular, there existed a minimal induction of IFNs from peripheral blood mononuclear cells derived from patients with SARS-CoV-1 and in MERS-CoV-infected ex vivo respiratory tissues [32,33]. Of note, our work was performed with a GFP tagged overexpression construct, therefore, the results need to be assessed along with viral infection. It will be important to confirm whether SARS-CoV-2 N protein would promote virus replication by blocking TRIM25-mediated IFN production, which was shown in contexts of SARS-CoV-1 infection [23], demonstrating the relevance of our findings in the context of SARS-CoV-2 infection in near future. 

Several SARS-CoV-2-encoded proteins, such as nsp1, nsp7, nsp15, papain-like protease, M, ORF3b, ORF6, and ORF9b, were found to inhibit the activation of IFN-mediated innate immune responses [7]. The molecular mechanisms by which SARS-CoV-1 N protein acts as an IFN antagonist have been examined by several groups [23,31,34]. In particular, Hu et al. demonstrated that the N proteins of SARS-CoV-1 targets TRIM25, thereby suppressing TRIM25-mediated RIG-I activation by ubiquitination [23]. Similarly, an inhibitory role of N protein was found in MERS-CoV in that N protein blocked RIG-I-induced type I and III IFN production via disrupting RIG-I ubiquitination by TRIM25 [35]. These findings highlight the inhibitory role of N protein of MERS-CoV and SARS-CoV-1 in the IFN pathways. In accordance with these data, we also found that the overexpression of SARS-CoV-2 N inhibited poly (I:C) or RNA virus-triggered activation of type I and III IFNs via interfering with the RLR pathway. In luciferase reporter assays, the overexpression of SARS-CoV-2 N protein significantly inhibited IFVdelNS1 or ZIKV (MR766)-triggered activation of type I and III IFNs in A549 cells (Figure 4A,B).

RLRs are major cytoplasmic sensors which drives IFN production and subsequent ISGs to control viral infection via detecting viral RNA ligands [36]. Viral recognition by RLRs can lead to TBK1-dependent activation of a transcription factor, IRF3, which upon activation, is translocated to nucleus for the induction of type I IFNs to trigger host antiviral innate immune responses [37]. Upon virus infection, IRF3 is phosphorylated at its C-terminus by TBK1 and IκB kinase ε, and is translocated into the nucleus as a dimer [38]. This subsequently initiates the transcription of target genes, including IFN-β [39]. Our results suggest that SARS-CoV-2 N-mediated inhibition of TBK1–IRF3 interaction, IRF3 phosphorylation, and nuclear translocation of IRF3 are possibly due to the consequences of interrupting the interaction between RIG-I and TRIM25. Furthermore, Figure 4F demonstrates the ability of SARS-CoV-2 N to interact with multiple RLR pathway molecules and interfere with their signaling.

Although SARS-CoV-2 has a genomic structure similar to that of SARS-CoV-1 or MERS-CoV, its clinical presentations are very different, which may be attributed to its different virulence mechanisms [2]. In summary, we characterized that the SARS-CoV-2 N protein is a major viral determinant for negative regulation of the RLR pathway and IFN production and signaling, demonstrating an effective yet unique method utilized by SARS-CoV-2 to evade and suppress host immunity. This observation expands our current understanding of the evasion strategies utilized by SARS-CoV-2 to antagonize the antiviral responses of the host.

## Supplementary Materials

The following are available online at https://www.mdpi.com/2073-4409/10/3/530/s1, Figure S1: HEK293T cells in 6-well plates were transfected with RIG-I-FLAG or MAVS-MYC plasmids along with empty vector (EV) with GFP tag (EV-GFP) or SARS-CoV-2 N-GFP (N-GFP) plasmids. After 24 h, cells were lysed and immunoprecipitated with indicated antibodies. Whole cell lysates (WCL) and immunoprecipitates were analyzed by immunoblot analysis.
