# Supplementary figures and images for: SARS-CoV-2 Nucleocapsid Protein Targets RIG-I-Like Receptor Pathways to Inhibit the Induction of Interferon Response
Source: Cells. 2021 Mar 2;10(3):530. doi: 10.3390/cells10030530 (PMC7999926; doi:10.3390/cells10030530)

RIG-I-FLAG + - +  
EV-GFP + - -  
N-GFP - + +

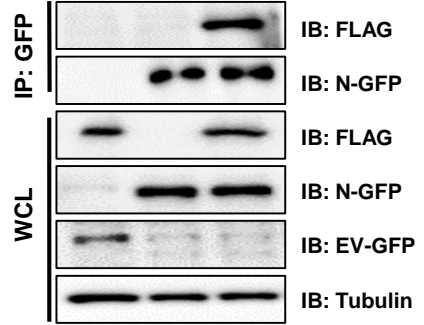

MDA5-HIS + - +  
EV-GFP + - -  
N-GFP - + +

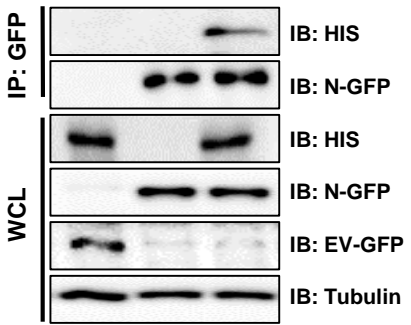

MAVS-MYC + - +  
EV-GFP + - -  
N-GFP - + +

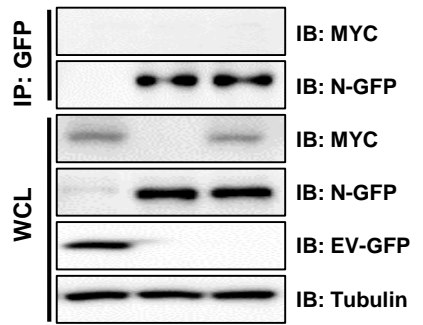

Supplement: Supplementary file 1 [file cells-10-00530-s001.pdf]
